# Observational case series on the clinical performance of the Variable Angle Clavicle Plate and Clavicle Hook Plate 2.7 systems: a study protocol

**DOI:** 10.3389/fsurg.2025.1694295

**Published:** 2026-01-08

**Authors:** Martin Jaeger, Julia Sußiek, Frank Beeres, Eben Carroll, Todd Conlan, Daniel Cunningham, Richard Arnhold, Simon Lambert

**Affiliations:** 1 Albert-Ludwigs-Universität Orthopädie and Traumatologie, Freiburg im Breisgau, Germany; 2 Department of Trauma-, Hand- and Reconstructive Surgery, University Hospital Muenster, Muenster, Germany; 3 Department of Orthopedic and Trauma Surgery, Lucerne Cantonal Hospital, Lucerne, Switzerland; 4 Faculty of Health Sciences and Medicine, University of Lucerne, Lucerne, Switzerland; 5 Atrium Health Wake Forest Baptist Orthopaedic Surgery, Winston-Salem, NC, United States; 6 Corewell Health, Department of Orthopedic Surgery, Grand Rapids, MI, United States; 7 Prisma Health Richland Hospital, Orthopaedic Trauma Surgery, Columbia, SC, United States; 8 Klinik Ottakring, Vienna, Austria; 9 The Princess Grace Hospital, HCA Healthcare UK, London, United Kingdom

**Keywords:** clavicle fractures, acromioclavicular joint dislocation, bone plates, osteosynthesis, plate prominence

## Abstract

**Background:**

Reoperation rates in clavicular fractures and acromioclavicular (AC) joint dislocations are high, often indicated for plate removal due to plate prominence and poor cosmesis. The recently designed 2.7 mm variable angle locking compression plate (VA-LCP®) Clavicle Plate and 2.7 mm VA-LCP® Clavicle Hook Plate (J&J MedTech) are intended to have an improved plate-to-bone fit, are precontoured, and have a low profile. Additionally, the Clavicle Hook Plate has a more anatomically adapted hook design to reduce the risk of painful osteolysis leading to earlier plate removals. All screw holes in these plates accept 2.7 mm screws. Primary objectives of this observational, post-marketing, prospective, multicenter case series are to gather early and mid- to long-term evidence on the clinical performance, safety, and usability of the 2.7 mm VA-LCP Clavicle Plates and 2.7 mm VA-LCP Clavicle Hook Plates, specifically to describe (1) safety, (2) clinical performance, (3) functional outcomes, (4) patient-reported outcomes (PROs) and (5) usability and surgeons' experience with the devices.

**Methods:**

Approximately 76 patients with clavicle fractures and primary surgical treatment with the 2.7 mm VA-LCP Clavicle Plate or 2.7 mm VA-LCP Clavicle Hook Plate will be enrolled prospectively over 15 months. Outcome measures include adverse events related/potentially related to the investigational device, clinical performance, functional outcomes (QuickDASH), PROs (PROMIS® upper extremity score, local pain, discomfort, and plate prominence), and radiographic outcomes. Data will be collected at baseline, 2w, 6w, 3m, 6m, 1y and 2y after surgery. The surgeons' experience with and perceived utility of the devices will be surveyed. Interim analysis will be performed when 30 patients per group have reached 6 months after surgery. Long-term outcomes will be evaluated using 2-year follow-up results. A Statistical Analysis Plan will be prepared before final analysis summarizing the descriptive statistics to be used.

**Discussion:**

The study described in this protocol on patients with clavicular fracture or AC joint dislocations undergoing surgical fracture fixation aims to gather first evidence on the clinical performance, safety, and usability of the 2.7 mm VA-LCP Clavicle Plates and 2.7 mm VA-LCP Clavicle Hook Plate Systems. This study will provide valuable data from a standard-of-care setting and may help design future comparative studies for relevant performance parameters.

**Clinical Trial Registration:**

https://clinicaltrials.gov/study/NCT04921865?term=NCT04921865%20&rank=1, identifier NCT04921865.

## 1 Introduction

There is still controversy and lack of consensus regarding not only surgical indications but also treatment of acute displaced clavicle shaft fractures and acute high-grade displaced acromioclavicular (AC) joint injuries (1–3). As surgical treatment has been increasingly used (1), higher complication and reoperation rates observed have led some to caution against overtreatment of displaced midshaft clavicular fractures (1, 4–6). After surgical treatment, reported reoperation rates in clavicular fractures range from 10%–84% (4–7) and up to 67% in AC joint dislocations (5). The need for plate removal is most often due to plate prominence and poor cosmesis due to the subcutaneous position of the clavicle and the bulky standard 3.5 mm implants. Galdi et al. examined whether using a lower profile 2.7 mm reconstruction plate would lead to better clinical outcomes and lower removal rates when compared with a 3.5 mm reconstruction plate in AO/OTA (Arbeitsgemeinschaft für Osteosynthesefragen/Orthopedic Trauma Association) type B clavicle fractures (8). The thinner 2.7 mm plates provided higher rates of cosmetic acceptability but did not demonstrate a statistically significant difference in reoperation rates for plate removal (8). Similarly, a recent retrospective comparative study also found that there was no statistically significant difference in rate of removal between 2.7 mm plates and 3.7 mm plates (9). Other studies have shown that clavicle fractures fixed with 3.5 mm reconstruction plates on the superior (subcutaneous) clavicle surface were more likely to exhibit plastic deformation, whereas constructs utilizing 2.7 mm reconstruction plates were more likely to fail by plate breakage (4).

The cost of clavicle fracture and AC dislocation treatment is high, ranging from a 2-year mean of US$27,635 [standard deviation (SD): US$68,173] to US$23,096 (SD: US$28,746), respectively (5), and up to GB£5000 for reoperations (7). Improving surgical methods and technologies could therefore reduce healthcare system costs through reduced unplanned reoperations and device removals. Additionally, predictable anatomic variance in clavicle morphology has been identified (10, 11), and understanding this variance could not only help improve the design and adaptation of clavicle implants, reducing the need for reoperations, but could also aid in implant selection (11).

The J&J MedTech 2.7 mm variable angle (VA) locking compression plate (VA-LCP®) Clavicle Plate System and the 2.7 mm VA-LCP® Clavicle Hook Plate System were designed to have an improved plate-to-bone fit. The plates have been pre-contoured taking into consideration the predictable anatomical variance of clavicle morphology and the correlation between individual patient height and clavicle length (11). The plates have a low profile to reduce construct prominence, soft tissue irritation, and related pain. All screw holes in these plates accept 2.7 mm screws, which is an important design adaptation in comparison to previous LCP 2.7/3.5 mm Clavicle Systems. Mechanical testing has shown that when the recommended screw configuration for each system is used, the 2.7 mm VA-LCP Clavicle Plates have higher construct static strength and equivalent construct fatigue strength compared with J&J MedTech 3.5 mm LCP Superior Clavicle plates (12).

The design rationale of the implant preferred pathway allows—for the first time—creation of a clavicle plate system matching the anatomy in a much higher number of cases. Further, the fixation strategy of the J&J MedTech 2.7 mm variable angle (VA) locking compression plate (VA-LCP®) Clavicle Plate System and the 2.7 mm VA-LCP® Clavicle Hook Plate System is new as the fracture-site centered-plate position is abandoned. Additionally, traditional clavicle hook plates, which were frequently used for the treatment of lateral clavicle fractures and acromioclavicular joint injuries, exhibit sub-optimal hook and plate geometry. Consequently, the hook is commonly directed towards the posterolateral corner of the acromion and does not match with the undersurface of the acromion (13), which might induce a raised point contact, osteolysis around the hook and/or subacromial impingement, and ultimately necessitate earlier implant removal. Both, the smaller plate design of the VA-LCP clavicle button hook plate, the anatomical plate design of the VA-LCP Clavicle Hook Plates and the anatomical hook design (10° anterior rotation; 16° inclination) are designed to address these issues. This new design will provide a better match of the hook to the undersurface of the acromion, and bring the tip of the hook more anterior, away from the posterolateral corner of the acromion.

This observational, post-marketing, investigator-initiated, prospective, multicenter case series aims to gather early and mid- to long-term evidence on the clinical performance, safety, and usability of the J&J MedTech 2.7 mm VA-LCP Clavicle Plate and 2.7 mm VA-LCP Clavicle Hook Plate Systems.

## 2 Methods and analysis

### 2.1 Study design and setting

This is an observational, post-marketing, prospective, multicenter case series on patients with clavicular fractures or dislocations of the AC joint undergoing surgical fracture fixation with either the 2.7 mm VA-LCP Clavicle Plate and 2.7 mm VA-LCP Clavicle Hook Plate Systems (J&J MedTech, Zuchwil, Switzerland). Table 1 summarizes study sites currently included. Currently, seven sites are participating in the study. Ethics approval has been obtained for six sites and is pending for one site. Thus far, 43 patients have been enrolled, of whom 42 meet the eligibility criteria.

**Table 1:** Current participating sites.

Name	Country	Region
Klinik Ottakring, Vienna	Austria	Europe
Universitätsklinikum Freiburg, Freiburg	Germany	Europe
Universität Münster, Münster	Germany	Europe
Prisma Health Richland Hospital, Colombia	USA	North America
Atrium Health Wake Forest Baptist, North Carolina	USA	North America
Corewell Health, Grand Rapids, Michigan	USA	North America
Luzerner Kantonsspital, Lucerne	Switzerland	Europe

### 2.2 Objectives

The objectives are to collect early and mid- to long-term evidence on the clinical performance, safety, and usability of the J&J MedTech 2.7 mm VA-LCP Clavicle Plate and 2.7 mm VA-LCP Clavicle Hook Plate System, specifically (1) safety, (2) clinical performance, (3) functional outcomes, (4) patient-reported outcomes (PROs), and (5) usability and surgeons' experience with the devices.

### 2.3 Study procedures

All treatments will be performed per local standard of care (SoC). Baseline information, treatment details, and outcomes will be collected in a customized, searchable database (REDCap Cloud Electronic Data Capture system (https://www.redcapcloud.com/) (14, 15). Post-treatment care and follow-up visits will be conducted according to standard procedures at participating sites but will take place within predefined time windows; if the 12-month post-hook plate removal visit is not foreseen at the participating site, the visit can take place remotely. Radiographic images will only be taken if part of the local SoC.

#### 2.3.1 Inclusion criteria

▪ Patients aged ≥18 years
▪ Unilateral clavicle injuries are considered for inclusion in the study. The patient must be undergoing primary surgical treatment within 21 days of injury using either of the following devices according to the manufacturer's instructions for use:
  ○ 2.7 mm VA-LCP® Clavicle Plates are used for fixation of clavicle bone fragments. Hereinafter referred to as Clavicle Plates
  ○ 2.7 mm VA-LCP® Clavicle Hook Plates are used for fixation of lateral clavicle fractures and dislocations of the acromioclavicular joint. Hereinafter referred to as Clavicle Hook Plates
▪ Expected ability to attend postoperative follow up (FU) visits
▪ Ability to provide informed consent

#### 2.3.2 Exclusion criteria

▪ Contraindications to the Clavicle Plates
  ○ Stable clavicle fractures, i.e., fracture which is not indicated for internal fixation
  ○ Fixation of sternoclavicular joint
  ○ Systemic infection or infection localized to the site of the proposed implantation
▪ Contraindications for the Clavicle Hook Plates
  ○ Stable lateral clavicle fractures, i.e., fracture which is not indicated for internal fixation
  ○ Fixation of sternoclavicular joint
  ○ Systemic infection or infection localized to the site of the proposed implantation
▪ Concomitant nerve or vessel injury
▪ Polytrauma (Injury Severity Score ≥16)
▪ Body mass index ≥40 kg/m^2^
▪ Uncontrolled severe systemic disease or terminal illness
▪ Intraoperative decision to use other implants is taken

#### 2.3.3 Recruitment

The recruitment period will be 15 months, during which at least 76 patients are planned to be enrolled. The participating sites will identify all eligible patients according to the defined inclusion and exclusion criteria. Eligibility assessment will be performed by the investigator and/or adequately trained research team member(s). The eligibility assessment includes but is not limited to an inquiry about the interest and willingness of the patient to participate in the study. Eligible patients identified after treatment (e.g., in case of an acute trauma followed by an emergency surgery) can be consented and enrolled at the latest by the time of discharge. Data collection for the study in the case report form (CRF) is not allowed without the documented agreement of the patient.

All consented patients included in the study will be allocated a unique patient number. Each site will keep an identification list linking the patient number to personal information. The identification list is always kept in a safe and locked place. Sites are not allowed to share the identification list with any third party except for the sponsor representative, legal authorities, and EC/IRB who may access the identification list during on-site monitoring/auditing performed on site.

All consented patients should be followed up within the study for a maximum of 2 years, except if their study participation is prematurely terminated.

#### 2.3.4 Data collection

**Figure 1:**
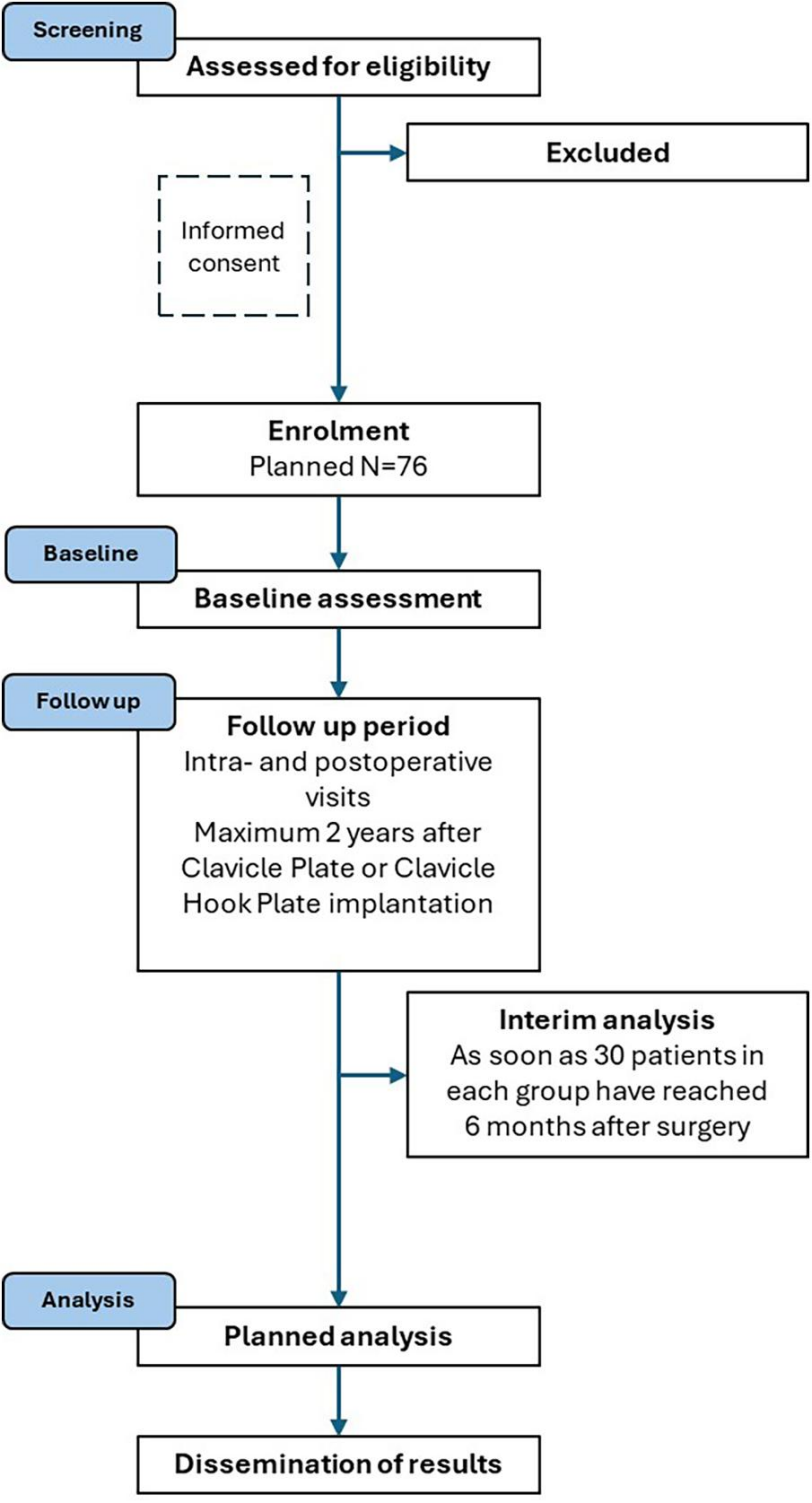
Planned patient flow (adapted from STROBE).

Tables 2, 3 show data to be recorded for Clavicle Plates and Clavicle Hook Plates. Figure 1 presents the planned patient flow.

**Table 2:** Study schedule for patients with clavicle plates (lateral, shaft, and medial).

Assessment parameters	Pre- intra- and postoperative visits*							
	Visit 1	Visit 2	Visit 3	Visit 4	Visit 5	Visit 6	Visit 7^#^	Visit 8^#^
	Screening/preoperative	Intraoperative (Day 0)	2 (±1) weeks	6 (±1) weeks	3 months (±2 weeks)	6 (±1) months	12 (±1) months	24 (±2) months
Patient information/consent^1^	X							
Eligibility^1^	X							
Patient characteristics	X							
Surgical details		X						
Adverse events		X	X	X	X	X	X	X
Device deficiencies		X	X	X	X	X	X	X
Bone healing				X	X	X	X	X
Surgeons’ evaluation		X						
QuickDASH	X^2^		X	X	X	X	X	X
Patient-reported outcomes^3^								
PROMIS Upper Extremity	X^2^		X	X	X	X	X	X
Local pain			X	X	X	X	X	X
Local discomfort			X	X	X	X	X	X
Plate prominence			X	X	X	X	X	X
Implant removal^π^			X	X	X	X	X	X
Images (x-ray/CT scan)^4^	X	X	X	X	X	X	X	X

CT, computed tomography.

* All postoperative follow-up visits with the defined time windows are calculated from the day of surgical treatment (i.e., Day 0).

# Visit can be done remotely.

π If applicable.

1 Informed consent may be obtained after treatment. No data can be collected in the electronic case report form prior to consent. See section 2.3.3.

2 This is the baseline assessment and refers to the preinjury status.

3 Can be assessed remotely.

4 Images will be taken only if it is part of the local standard of care.

**Table 3:** Study schedule for patients with clavicle hook plates.

Assessment parameters	Pre- intra- and postoperative visits								
	Visit 1	Visit 2	Post-implantation visits*			Visit 6^£^	Post hook plate removal visits**		
			Visit 3	Visit 4	Visit 5^£^		Visit 7**	Visit 8**	Visit 9^**^^,^^#^
	Screening/ preoperative	Intraoperative (Day 0)	2 (±1) weeks	6 (±1) weeks	3 months (±2 weeks)	Implant removal surgery (Day 0′)	6 (±1) weeks post-removal	3 months (±2 weeks) post-removal	12 (±1) months post-removal
Patient information/consent^1^	X								
Eligibility^1^	X								
Patient characteristics	X								
Surgical details		X							
Adverse events		X	X	X	X	X	X	X	X
Device deficiencies		X	X	X	X	X			
Bone healing^π^				X	X	X	X	X	X
Joint healing							X	X	X
Surgeons’ evaluation		X							
QuickDASH	X^2^		X	X	X	X^5^	X	X	X
Patient-reported outcomes^3^									
PROMIS upper extremity	X^2^		X	X	X	X^5^	X	X	X
Local pain			X	X	X	X^5^	X	X	X
Local discomfort			X	X	X	X^5^	X	X	X
Plate prominence			X	X	X	X^5^			
Implant removal						X			
Intraoperative shoulder stability						X			
Images (x-ray/CT scan)^4^	X	X	X	X	X	X	X	X	X

CT, computed tomography.

* All postoperative follow-up visits with the defined time windows are calculated from the day of surgical treatment (i.e., Day 0).

** All post-removal follow-up visits with the defined time windows are calculated from the day the hook plate is removed (i.e., Day 0′).

£ Whichever occurs first.

π If applicable.

# Visit can be done remotely.

1 Informed consent may be obtained after treatment. No data can be collected in the case report form prior to consent. See Section 2.3.3.

2 This is the baseline assessment and refers to the preinjury status.

3 Can be assessed remotely.

4 Images will be taken only if it is part of the local standard of care.

5 Refers to the status prior to the implant removal surgery.

Data from participating patients are documented in electronic CRFs and captured using the REDCap Cloud Electronic Data Capture system (https://www.redcapcloud.com/) (14, 15) to which only authorized personnel have access. After termination of the study, each site will receive an electronic copy of its own data. Based on the risk analyses, no data monitoring committee is planned. Regular data monitoring and cleaning will be performed to ensure data accuracy.

##### 2.3.4.1 Baseline information

Demographic data include sex and year of birth, height, weight, and body mass index. Concomitant diseases will be assessed using the Charlson Comorbidity Index (16, 17).

##### 2.3.4.2 Injury details

Injury details include the date of injury, side of injury, and type of injury. Clavicle fracture will be recorded using the AO/OTA classification (18) and the Robinson classification (19), and AC joint dislocation using the Rockwood classification (20).

##### 2.3.4.3 Treatment details

Surgical details include:

▪ Date of surgery when the fixation with a Clavicle Plate/Clavicle Hook Plate is performed.
▪ Surgical time (skin-to-skin time)
▪ Plate details (type of plate and size and material for Clavicle Plates, and type of plate, hook depth and material for Clavicle Hook Plates)
▪ Screw details
▪ Adjunctive fixation (osseous cerclage suture, direct ligament suture, internal brace, ligament augmentation for primary repair, suture anchors, muscular reattachment or other)
▪ Fluoroscopy time (in min)

##### 2.3.4.4 Imaging data

Each site will take images per local SoC (routine) procedures. All images will be deidentified and sent to the sponsor.

##### 2.3.4.5 Follow-up schedule

Follow-up visits with defined time windows will be calculated from either the treatment day (Day 0) or the day of implant removal (Day 0′). All patients will be followed up for a maximum of 2 years after Clavicle Plates or Clavicle Hook Plates implantation. To ensure fair comparisons of treatment outcomes, the FU period is defined as follows:

▪ For Clavicle Plates (shaft, lateral or medial), Day 0 is defined as the day of surgical treatment with the plate.
▪ For Clavicle Hook Plates, Day 0 is defined as the day of surgical treatment with the plate. Scheduled FU visits will be calculated from Day 0 until the plate is removed. After the plate is removed, the subsequent FU visits will be calculated from the day of removal (Day 0′)

#### 2.3.5 Unscheduled visits

Unscheduled visits outside of the defined timepoints may take place any time during the study in cases of medical emergency or if deemed appropriate for patient care. No data will be recorded in the eCRF except for any device or treatment related adverse events (AEs). Any images taken will be sent to the sponsor, and a drop out form will be completed if a patient drops out.

#### 2.3.6 Termination of participation

Patient participation in the study may terminate for reasons such as withdrawal of informed consent, failure to commence treatment within the study, post enrolment screening failure, investigator's discretion (e.g., patient noncompliance), loss to FU, death, and other unspecified reasons. Detailed information explaining the reasons for termination will be recorded in a dropout form. For these patients, the collected data will be censored at the day of termination and may be used in the analysis.

#### 2.3.7 Outcome measures

Outcome measures will be assessed according to the schedule in Tables 2, 3.

##### 2.3.7.1 Adverse events

Safety will be assessed by the incidence of AEs that are related, probably related, or possibly related to the investigational device (i.e., adverse device effects [ADEs]) or the procedure. The events collected are described in the Supplementary Material.

##### 2.3.7.2 Clinical performance

Outcome measures related to clinical performance to be recorded are a subset of safety-related parameters including:

▪ Incidence of complaints related to plate prominence or discomfort
▪ Incidence of complaints related to local pain
▪ Reoperation rate due to AE probably or possibly related to the investigational device
▪ Fracture union rate
▪ Rate of stable AC joint healing (if applicable) at one-year post-removal, clinically assessed

##### 2.3.7.3 Bone and joint healing

Bone healing and AC joint healing (in case of Clavicle Hook Plates only) will be assessed by the treating surgeon following local SoC. The global clinical assessment of the patient's status according to the surgeon's judgement will be documented as not assessable, no signs of healing, healing in progress (normal but slow), healing in progress (normal) or healed. In patients receiving Clavicle Hook Plates, the AC joint healing status will be assessed only after the plate has been removed. The global assessment from the surgeon on the joint healing will be documented as: not assessable, not healed, partially healed, or healed.

##### 2.3.7.4 Surgeons' evaluation

Surgeons will be asked to evaluate their subjective experience with the Clavicle Plate/Clavicle Hook Plate system using the following two 5-point Likert scale questions:

Surgical time (skin-to-skin) required for fracture reduction and fixation (answers ranging from “much less time than with other implants” to “much more time than with other implants”)
Surgeons' impression of the device fit (answers ranging from “implant fitted well, no contouring required” to “implant required contouring medially and laterally”).

##### 2.3.7.5 Functional outcomes

Functional outcomes used in this study include the Disabilities of the Arm Shoulder and Hand (DASH) short form (QuickDASH), an occupational health and disability questionnaire (officially translated in 54 languages) (21, 22). Scaling ranges from 0 (least disability) to 100 (most disability). The minimal detectable change (MDC) at the 95% confidence level (MDC95) for QuickDASH has a mean of 18 (range: 16–20), though some indicators place the minimal clinically important differences at or below this threshold.

##### 2.3.7.6 Patient-reported outcomes

PROs used in this study are the Patient-Reported Outcomes Measurement Information System (PROMIS®) (23), a set of person-centered measures that evaluate and monitor physical, mental, and social health in adults and children. The PROMIS® Physical Function Upper Extremity Short Form 7a v2.0 (2018) consists of seven questions to evaluate activities requiring use of the upper extremity (shoulder, arm and hand).

Additional PROs including local pain, local discomfort and plate prominence will be assessed using an NRS. The local pain NRS (24, 25), scale ranges from 0 (no pain) to 10 (worst imaginable pain), where pain NRS ≥5 (severe pain) will be considered as an AE. The NRS for local discomfort related to the plate ranges from 0 (no discomfort) to 10 (maximum degree of discomfort imaginable). Patients reporting discomfort NRS ≥1 will be asked if the discomfort produced by the plate requires medical or surgical treatment. If such treatment is required, it will be considered an AE. The plate prominence NRS ranges from 0 (no prominence) to 10 (maximum level of prominence). Patients reporting plate prominence NRS ≥1 will be asked if they require medical or surgical treatment to address the prominence. If treatment is required, it will be considered an AE.

##### 2.3.7.7 Implant removal

The rate of implants removed during the study together with the reasons and timing of removal will be analyzed. Data collected include date of surgery and reason for removal. If the implant is removed due to an AE or device deficiency, this will be documented on the specific form. In patients with Clavicle Hook Plates, upon plate removal the stability of the shoulder ligaments will be assessed intraoperatively. The AC joint stability will be assessed as stable, partially unstable, or unstable.

##### 2.3.7.8 Device deficiencies

All device deficiencies will be reviewed, determined and documented in writing whether they could have led to a serious ADE.

##### 2.3.7.9 Radiographic outcomes

Radiological images will be taken per local SoC, and images will be transmitted to the sponsor. The central reader (a medical specialist with the appropriate training [SL]) will receive the images in a blinded way (no personal patient data except gender), and central analysis will be done at the interim analysis and at the end of the study. The following parameters will be assessed at both time points:

Screw density and distribution
Quality of reduction
AC and CC distance
Bone union
Osteolysis (of the acromion)
Heterotopic ossification (in any site)

### 2.4 Statistical analysis

This is an observational case series aiming to collect data on clinical performance, safety and usability of Clavicle Plates and Clavicle Hook Plates. Therefore, there is no formal statistical hypothesis in this study.

#### 2.4.1 Sample size determination

The study objective is to describe long term outcomes (status following healing of the related clavicle injuries) in a minimum of 30 patients with shaft fractures treated with Clavicle Plates and 30 patients with lateral fractures/AC dislocations treated with Clavicle Hook Plates. Assuming a 20% dropout rate at two years, the study will need to enroll 76 patients (38 patients per group).

#### 2.4.2 Statistical analysis

A detailed Statistical Analysis Plan (SAP) will be prepared before the final analysis. Simple summary statistics will be generated for baseline characteristics (demographics, somatometric parameters, comorbidities, and type of injuries) and outcomes recorded at scheduled FU assessments. Categorical variables will be summarized using frequency and percentage for each category; continuous variables will be summarized using mean, standard deviation, median, interquartile range, and minimum and maximum values. Continuous variables with both baseline and FU measurements available will also be summarized as changes between baseline and FUs at the individual patient level.

AEs will be reported both at patient level and AE level. The risk estimates will be presented along with the 95% Clopper-Pearson confidence interval. When calculating the AE rates, the denominator will be the full analysis population, irrespective of dropouts during FU. Sensitivity analyses will be conducted excluding patients who dropped out of the study and had not experienced an AE. For patients treated with Clavicle Plates, the risk of experiencing any ADE within 24 months after surgery will be reported. For the calculation of the ADE rate, only ADEs with onset before or within the upper visit window of the 24 months visit will be included. For patients treated with Clavicle Hook Plates, the risk of experiencing any ADE between surgical treatment and 12 months after removal of the Clavicle Hook Plate will be reported. Separate sub analyses on the risk of experiencing an ADE between surgery and Clavicle Hook Plate removal and within 12 months after the Clavicle Hook Plate removal will be performed. Separate ADE rates for the two FU periods including only ADEs with onset within the upper boundary of the visit window of the corresponding FU period will be calculated. For incidences of complaints related to plate prominence or discomfort and to local pain, incidence rates will be calculated, and summary statistics presented, including the number and percentage of patients with complaints related to plate prominence or discomfort (defined as NRS ≥1) and local pain (defined as NRS ≥3). For the reoperation rate due to ADE, fracture union rate, rate of stable AC joint healing at one-year post-removal, the full analysis population will be used as the denominator. Descriptive analyses on the rate of implants removed and the reasons for implant removal will be conducted. Simple summary statistics (i.e., numbers and percentages) will be produced for each bone healing category. QuickDASH, will be measured repeatedly during the FU period and analyzed using mixed effects models for repeated measures (MMRM). PROs also measured repeatedly during the FU period will likewise be analyzed using MMRM. For the surgeons' evaluation, simple summary statistics including numbers and percentages will be produced for each category.

An interim analysis will be performed as soon as 30 patients in each group have reached the timepoint of 6 months after surgery. The main purpose of the analysis is to ensure safety, while the potential implications could include stopping the study, amending the protocol, or early dissemination of results, depending on the analysis of the study. Definition of the analysis populations will be specified in the SAP.

The planned statistical analysis will allow for repeated analyses on the per-protocol population in cases of major protocol deviations, as well as sensitivity analyses excluding patients who dropped out of the study and had not experienced an AE. Some degree of missing data is expected. Patients may withdraw from the study prior to completion of the intended FU for reasons such as death, severe intercurrent illness, withdrawal of consent, and loss to follow-up. In case of missing data a decision will be made whether special analysis methods are required to handle missing data on an individual basis for each endpoint (as defined in the SAP). This decision will be made based on the extent of the missing data and the likelihood of bias occurring because of the missing data, and the importance of the endpoint. Protocol deviations will be collected in the study database in a specific protocol deviation form and listed in the final analysis. All protocol deviations will be reviewed during the data review meeting prior to final analysis.

## 3 Discussion

This study protocol describes an observational, post-marketing, multicenter case series to collect first evidence on the performance, safety and usability of the J&J MedTech 2.7 mm VA-LCP Clavicle Plate and 2.7 mm Clavicle Hook Plate Systems in a clinical setting. This system is newly developed for the surgical treatment of clavicle fractures and AC joint dislocations—all fixation screws are 2.7 mm in diameter. The system was designed for optimal surface contour fit and implant dimensions, without loss of implant or fixation strength when compared with standard 3.5 mm implants. Since this system has recently been launched on the market, an observational case series with a descriptive analysis is the most appropriate design to collect the first clinical evidence.

One main reason for reoperations after surgical treatment of clavicle fracture and AC joint dislocations is plate prominence (4–7), yet many patients request plate removal for various reasons. In the clinical community, differing opinions exist at both surgeon and country level on plate usage and removal (9, 26). Osteosynthesis with plates may be avoided due to the expected need for a reoperation, conversely plates might be removed as standard to prevent future complications (26). This impacts the overall cost effectiveness of the original surgical treatment using plates, as the cost for plate removal must also be considered (5, 7). Plate removal in asymptomatic cases is different from that for plate prominence, which may be associated with symptoms or complications. Thus, it is important to distinguish between plate prominence and plate removal as reasons for reoperation. Plate prominence is defined as symptomatic excessive subcutaneous prominence of that part of the plate aligned to the shaft or lateral clavicle cortex which may be related to poor fit of the plate to the clavicle, protruding plates or even screw head prominence, the latter of which has been overcome with locking screws or low-profile, non-locking screws (27). Further, plate prominence is influenced by the plate profile and by the fit between the plate and the bone (27). Plate prominence may lead to irritation of the soft tissues and may lead to pain (28), and is the most common reason for reoperation after clavicular shaft plate osteosynthesis (28). Additionally, mismatch of the orientation, angulation or length of the hook region of the clavicular hook plate with respect to the inferior surface of the acromion may cause intrusion into the subacromial space (leading to subacromio-deltoid bursitis and possible extrinsic rotator cuff attritional lesions) or, conversely, erosive attrition of the acromial periosteum and, eventually, bone (leading to bone pain, bone erosion, and possible bone fracture) (29, 30).

With the 2.7 mm VA-LCP Clavicle Plates and 2.7 mm Clavicle Hook Plates used in this study there is a downsizing of the implant/bone interface. As the first all 2.7 mm clavicle plate system it is important to be able to provide data on its clinical safety, but also important data on its clinical performance including complaints related to plate prominence or discomfort and PROs assessing these aspects.

In this study, a prospective, observational design is used which will allow an assessment of the 2.7 mm VA-LCP Clavicle Plates and 2.7 mm Clavicle Hook Plates when they are used per SoC, as the protocol does not influence the clinical decision-making process. MMRM will be used to analyze PROs and QuickDASH, allowing us to consider the within-subject correlations, and adjust for baseline characteristics and other covariates, improving estimate and inference precision. Additional analyses will be adjusted as appropriate, depending on their objectives and model assumptions. The list of confounders and the details of the adjustment methods will be fully specified in the Statistical Analysis Plan. The sensitivity analysis, the analysis restricted to per-protocol population, and the handling of missing data (if deemed necessary) will help assess whether the conclusions remain consistent when accounting for potential biases introduced by major protocol deviations, missing data, and/or patient dropout.

Data gathered from this study can help design comparative studies for relevant performance parameters in the future. Indeed, a limitation of this study is the absence of a control group, yet the results of this study will be useful in future comparative studies, providing a proven treatment arm which could be compared to different treatment options. Also, although this study does not specifically aim to assess cost-effectiveness, the evidence gathered on the need for reoperations may help drive hypotheses development for future studies focused in this area; additionally, a *post hoc* cost-benefit analysis might take place. This study does have some limitations. One limitation of the study protocol is the lack of patient involvement in its development; there may be other outcomes important to patients that are missing from the present study. As a prospective, observational case series without a control or comparator group, this study is limited in its ability to establish causal relationships or directly compare the investigational plates with other fixation methods. The relatively small sample size and potential loss to follow-up may reduce statistical power and generalizability of the findings. Additionally, generalizability to comorbid populations may be limited because the population included is generally healthy with patients with certain comorbidities and polytrauma being excluded. Outcomes rely partly on patient-reported measures, which may be subject to bias, particularly recall, reporting or social desirability biases which may affect how accurately patients describe their symptoms. The multicenter design may introduce variability in surgical technique and postoperative management. This also impacts variability in imaging acquisition as imaging acquisition follows the local standard of care; this limitation of multicenter observational studies will be mitigated using a central reviewer who receives the images in a blinded way and centrally analyses the images at both the interim and final analysis following standardized criteria. Lastly, as the study is multicenter, it is further subject to geographical variability; there may be differences in surgical expertise, health care systems or patient demographics that may influence the results obtained. Nevertheless, the multicenter collaborative effort will provide clinical and safety data from different geographic locations per SoC, providing initial knowledge to form the basis for future clinical research.

## 4 Ethics and dissemination

Ethics approval is obtained from the local ethics committee or institutional review board prior to patient enrolment (Corewell Health—West Institutional Review Board CHW-IRB-2025-0954; PRISMA HEALTH IRB 2248577-1; Ethik-Kommission Albert-Ludwigs-Universität Freiburg 24-1346-S1-AV; Ethikkommission Nordwest- un Zentralschweiz 2024-01581; Ethik Kommission Westfalen-Lippe 2024-207-f-S; Wake Forest University Health Sciences IRB IRB00104269; Ethikkommission der Stadt Wien EK 24-096-0824). The registry has been designed and implemented according to current valid international standards (ICH GCP and ISO 14155) and based on the ethical position of the Declaration of Helsinki, to ensure optimal protection of patient interests. For patient enrolment an informed consent process approved by the responsible ethics committee will be followed. It is intended that the results of this study shall be published in peer-reviewed journals. For the publication of the results of this study, all investigators will have access to all data generated and be involved in the decision to publish. This manuscript reflects the current protocol version V1.0 dated September 12, 2023.

## Supplementary material

Supplementary Data Sheet 1 Outcome measures - adverse events.
