# Supplementary material for: Observational case series on the clinical performance of the Variable Angle Clavicle Plate and Clavicle Hook Plate 2.7 systems: a study protocol
Source: Front Surg. 2026 Jan 8;12:1694295. doi: 10.3389/fsurg.2025.1694295 (PMC12823801; doi:10.3389/fsurg.2025.1694295)
Supplement: Supplementary Data Sheet 1 — Outcome measures - adverse events. [file Datasheet1.pdf]

Observational case series on the clinical performance of the Variable Angle Clavicle Plate and Clavicle Hook Plate 2.7 systems: a study protocol

Martin Jaeger, Julia Sußiek, Frank Beeres, Eben Carroll, Todd Conlan, Daniel Cunningham, Richard Arnhold, Simon Lambert

Outcome measures

Adverse events

The following events will be collected.

Soft tissue complications:

- Damage to neurovascular structures
- Local soft tissue irritation, erosion, or damage
- Local infection
- Coracoclavicular (CC) or AC ligament healing complications
- Subacromial impingement

Bone healing complications:

- Refracture/peri-implant fracture
- Osteomyelitis
- Osteolysis
- Subacromial surface erosion/fracture
- Delayed union (defined as absence of bone bridging of the fracture[s] gap[s] on at least three of four cortices on orthogonal radiographs taken at the 3-month FU), nonunion (defined as absence of bone bridging of the fracture[s] gap[s] on at least three of four cortices on orthogonal radiographs taken at the 6-month FU), or malunion (defined as a fracture which has healed in a non-anatomic position, whatever the translation [lineal, rotational, and axial] or angulation may be).
- Osteoarthritis

Mechanical failures:

- Fixation failure
- Loss of reduction
- Implant cut-out (through acromion)
- Hardware failure

Pain, discomfort, or prominence complications:

- Local pain numeric rating scale (NRS) ≥ 5, local discomfort due to the plate that requires medical or surgical treatment, or plate prominence that requires medical or surgical treatment.

Any other AEs judged to be ADEs or potential ADEs will also be collected.
